# The molecular structure, biological roles, and inhibition of plant pathogenic fungal chitin deacetylases

**DOI:** 10.3389/fpls.2023.1335646

**Published:** 2024-01-09

**Authors:** Johannes Mapuranga, Jiaying Chang, Hao Li, Yingdan Zhang, Ruolin Li, Lulu Song, Na Zhang, Wenxiang Yang

**Affiliations:** College of Plant Protection, Technological Innovation Center for Biological Control of Plant Diseases and Insect Pests of Hebei Province, Hebei Agricultural University, Baoding, China

**Keywords:** plant pathogenic fungi, chitin deacetylases, molecular structure, biological roles, inhibition

## Abstract

Chitin/polysaccharide deacetylases belong to the carbohydrate esterases family 4 (CE4 enzymes). They play a crucial role in modifying the physiochemical characteristics of structural polysaccharides and are also involved in a wide range of biological processes such as fungal autolysis, spore formation, cell wall formation and integrity, and germling adhesion. These enzymes are mostly common in fungi, marine bacteria, and a limited number of insects. They facilitate the deacetylation of chitin which is a structural biopolymer that is abundantly found in fungal cell walls and spores and also in the cuticle and peritrophic matrices of insects. The deacetylases exhibit specificity towards a substrate containing a sequence of four GlcNAc units, with one of these units being subjected to deacetylation. Chitin deacetylation results in the formation of chitosan, which is a poor substrate for host plant chitinases, therefore it can suppress the host immune response triggered by fungal pathogens and enhance pathogen virulence and colonization. This review discusses plant pathogenic fungal chitin/polysaccharide deacetylases including their structure, substrate specificity, biological roles and some recently discovered chitin deacetylase inhibitors that can help to mitigate plant fungal diseases. This review provides fundamental knowledge that will undoubtedly lead to the rational design of novel inhibitors that target pathogenic fungal chitin deacetylases, which will also aid in the management of plant diseases, thereby safeguarding global food security.

## 1 Introduction

Global food security is continuously threatened by plant diseases which cause severe economic losses. Several plant diseases have been reported to be caused by both soil-borne and airborne phytopathogenic fungi and the management of these diseases is still complicated (Fones et al., 2020). During the battle between host plants and fungal pathogens, plants utilize cell surface-localized pattern recognition receptors (PRRs) to detect potential threats in the apoplast environment. These receptors recognize pathogen-associated molecular patterns (PAMPs) and plant-derived damage-associated molecular patterns (DAMPs) as danger signals (Jones and Dangl, 2006; Monaghan and Zipfel, 2012). These signals serve as stimuli for the activation of the plant immune system, resulting in the translocation of a combination of plant defense molecules to the apoplast to deter pathogen invasion (Doehlemann and Hemetsberger, 2013). The fungal cell wall serves a crucial function in preserving cellular integrity and adapting to intricate and dynamic environmental conditions. The effectiveness of a fungal pathogen invasion on a host is contingent upon its ability to circumvent the plant’s intrinsic immune system, which identifies the conserved elements of the fungal cell wall, such as chitin (Dai et al., 2021). Phytopathogenic fungi have undergone adaptive modifications in their cell wall composition in response to co-evolutionary pressures, thus enabling them to effectively combat infections. One of the primary strategies is the safeguarding of PAMPs from identification by host receptors. Well adapted pathogens overcome this defensive barrier by using strategies such as concealing their cellular surfaces, isolating PAMPs, and altering the glycans present in their cell surfaces. During infection, fungi conceal chitin in their cell walls by either overlaying it with additional polymers or subjecting it to deacetylation facilitated by an enzyme known as chitin deacetylase (CDA), resulting in the formation of chitosan. Chitosan cannot be recognized by chitinases, implying that, it is a poor substrate for chitinases (Brunner et al., 1998; Sánchez-Vallet et al., 2015), therefore it can suppress the host immune response triggered by pathogens and facilitate colonization (Stegrist and Kauss, 1990).

Chitin deacetylases (CDAs; EC 3.5.1.41) are a class of enzymes that catalyze the hydrolysis of the acetamido group in N-acetylglucosamine (GlcNAc) units found in chitin to produce chitosan. Chitin deacetylases are classified under family 4 of carbohydrate esterases (CE4) in accordance with the CAZy categorization system (www.cazy.org) (Lombard et al., 2014). The genomes of chitin-containing fungi exhibit a significant presence of putative members belonging to the CE4 family, suggesting their potential significance in several biological processes (Xie et al., 2020). Active deacetylases within this family possess five conserved sequence motifs, which contain conserved aspartic acid and histidine residues, as well as a binding site that is crucial for the catalytic activity of a metal ion (Blair et al., 2005; Puchart et al., 2006; Taylor et al., 2006; Baker et al., 2007; Andrés et al., 2014; Liu et al., 2023). CDAs exhibit a specific affinity for a substrate containing a sequence of four GlcNAc units. Within this sequence, one GlcNAc unit undergoes deacetylation, resulting in a chitosan product with a more uniform deacetylation pattern compared to chitosan produced through the treatment of chitin with hot sodium hydroxide (Zhao et al., 2010). The first chitin deacetylase (MrCDA) to be identified, purified, and characterized was from the fungus *Mucor rouxii* (Araki and Ito, 1975; Kafetzopoulos et al., 1993), and subsequently, various studies reported CDAs from different fungi. These enzymes exhibit five conserved motifs, a conserved NodB homology domain, and six distinct loops. In addition to the conserved NodB domain, several CDAs also possess chitin-binding domains which facilitate the deacetylation of chitin by removing acetyl groups (Zhao et al., 2010). CDAs exhibit a wide range of features and ideal functioning conditions, mirroring the diversity found in their sources including bacteria, fungi, and insects, and they typically have a molecular weight spanning from 12 to 150 kilodaltons (kDa). The optimal temperature range for the activity of CDAs is often between 30 to 60°C, and their optimal pH levels range from 4.5 to 12 (Bonin et al., 2020). Certain CDAs comprise of catalytic domains fused with carbohydrate binding modules which appear to augment the accessibility of chitin chains to the catalytic domain which in turn, leads to a modest improvement in deacetylase activity (Hoßbach et al., 2018).

CDAs are secreted at a distinct time interval that aligns with their specific biological functions. Fungal plant pathogens secrete CDAs during the early infection and initial growth stage in the host plant to evade the plant defense mechanisms activated by plant chitinases (Zhao et al., 2010). For example, it was established that the secretion of an extracellular CDA from *Colletotrichum lindemuthianum* (*C. lindemuthianum*) occurred solely during the penetration of fungal hyphae into plants (Tsigos and Bouriotis, 1995). The expression of CDAs is highly induced during the early stages of infection. It was proposed that when the cell wall chitin or the chitooligosaccharides produced by chitinases undergo partial deacetylation, it leads to the formation of partially deacetylated oligomers that are not easily recognized by plant receptors, which in turn reduces the activation of plant defense responses (Cord-Landwehr et al., 2016). Notably, gaining an in-depth understanding of CDAs and peptidoglycan deacetylases as potential targets for the advancement of antimicrobial agents has garnered attention, highlighting the need to enhance our comprehension of the catalytic mechanism and structure of these enzymes (Vollmer and Tomasz, 2002). To date, a considerable number of reviews about CDAs from various organisms have been published, including comprehensive discussions on the classification, configuration, catalytic mechanism, and their potential applications (Aragunde et al., 2018; Grifoll-Romero et al., 2018; Bonin et al., 2020). However, no review has specifically focused on plant pathogenic fungal CDAs and their inhibitors. Therefore, this review discusses the plant pathogenic fungal CDAs, their structure including their 3-dimensional (3D)-structure, conserved motifs, catalytic mechanism, and substrate specificity. The biological roles of these CDAs will also be discussed as well as the recently discovered chemicals (inhibitors) that attenuates plant fungal disease, suggesting that they can help to control plant fungal diseases.

## 2 Structural and sequence features of plant pathogenic fungal CDAs

### 2.1 3D and crystal structures

The first 3D-structures of the CE4 family were determined by X-ray crystallogy and this includes the crystal structure of *Bs*PdaA (PDB 1W17), and *Sp*PgdA (PDB 2C1G) a peptidoglycan deacetylase from *Bacillus subtilis* and, *Streptococcus pneumoniae*, respectively (Blair and van Aalten, 2004; Blair et al., 2005). This was then followed by the determination of the first crystal structure of a CDA from *C. lindemuthianum, Cl*CDA (PDB 2IW0) which had a high degree of conservation in the structure of His-His-Asp zinc-binding triad and catalytic active sites (Blair, 2006). Currently, all the known CE4 family CDAs have a catalytic domain characterized by a (β/α)_8_ or (β/α)_7_ barrel and one of the αβ repeats of regular TIM barrels may lack the ability to form a groove for the binding of the extended polymer substrate (Nakamura et al., 2017). The central core consists of seven or eight parallel β-strands that form a highly distorted β-barrel shape, which is encircled by α-helices. The β-barrel is adorned with a sequence of loops, which constitute the main component of the carbohydrate binding pocket. The loops vary in length, sequence composition, topology, and dynamics (Aragunde et al., 2018). The presence or absence of these loops around the catalytic sites determine the substrate specificity of different CDAs and the flexibility of these loops is also significant (Andrés et al., 2014). Long loops have a high degree of specificity due to their buried active sites and the presence of narrow binding pockets in their structure. Single-site deacetylations are due to the substrate’s limited ability to bind in specific ways. Short loops show wide specificity and have a binding cleft that is both flat and open. Multiple chain mechanisms of deacetylation are attributed to the ability of the structure to slide along in various modes. The precise mechanism underlying the generation of specific patterns for all CDAs remains poorly understood.

**Figure 1:**
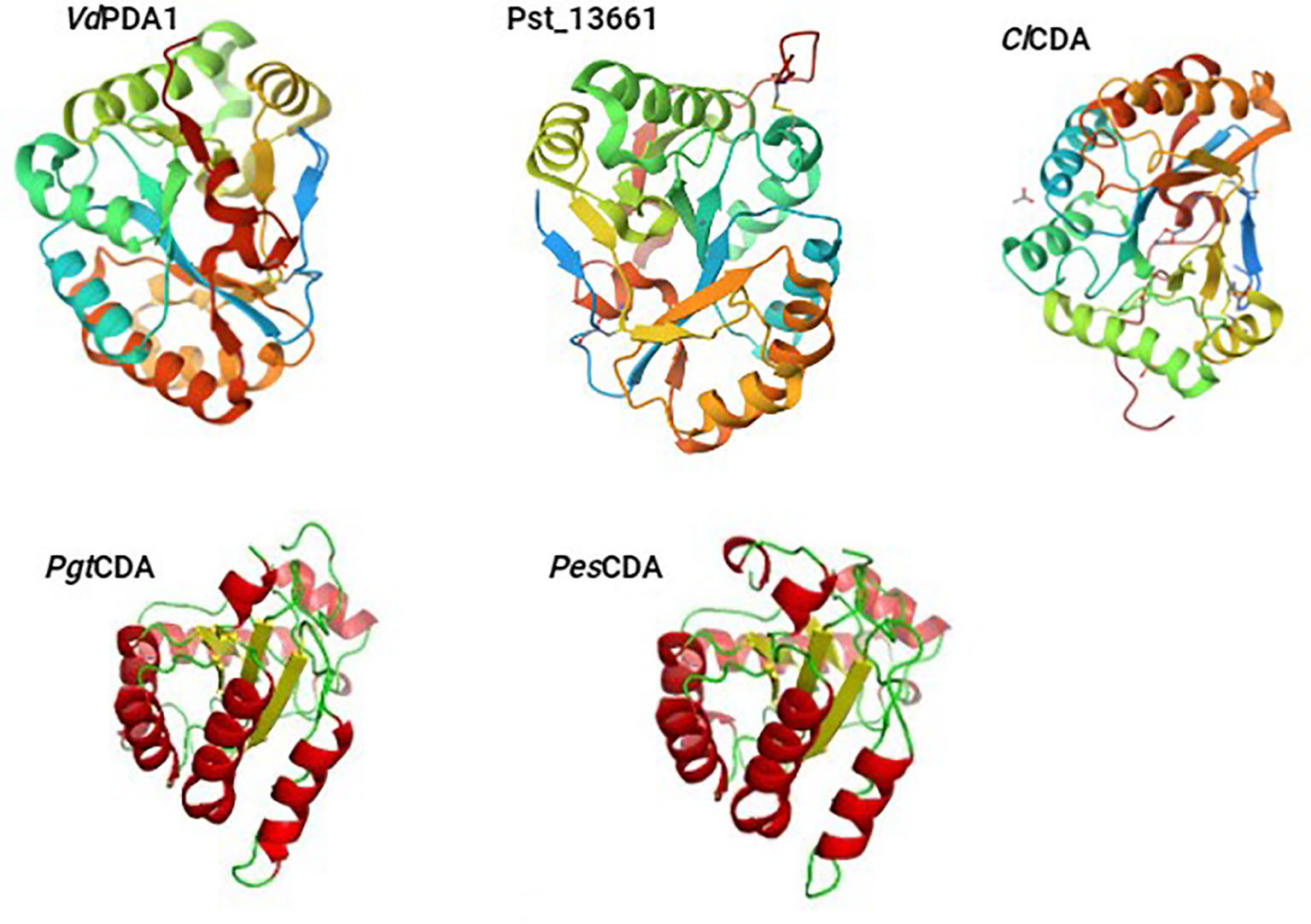
The 3D structures of the plant pathogenic fungal CDAs, either already reported with structures determined by X-ray crystallography (*Vd*PDA1, Pst_13661, and *Cl*CDA) or generated by SWISS MODEL and PyMOL (*Pgt*CDA and *Pes*CDA).

CE4 enzymes exhibit notable topological variations, with some enzymes having their N/C-termini positioned on the same side of the barrel (e.g., *Cl*CDA), while others have their N/C-termini situated on opposing ends (e.g., *Sp*PgdA and *Bs*PdaA) (Blair, 2006). Some of the 3D structures of the plant pathogenic fungal CDAs have been already reported with structures either determined by X-ray crystallography (*Cl*CDA) or generated by SWISS MODEL and PyMOL (*Pgt*CDA and *Pes*CDA) (**Figure 1**). Recently Liu and colleagues obtained crystal structures of CDAs *Vd*PDA1 (PDB 8HFA) and Pst_13661 (PDB 8HF9) from *Verticillium dahliae* (*V. dahliae*) and *Puccinia striiformis* f. sp. *tritici* (*Pst*), respectively (**Figure 1**). These structures indicated that both CDAs consist of a substrate-binding pocket that is similar, as well as an His-His-Asp triad that serves to coordinate a transition metal ion. It was also established that both *Vd*PDA1 and Pst_13661 have short and open-ended substrate-binding grooves. In terms of configuration, they both form a typical (β/α) fold (Liu et al., 2023). Having a root mean square deviation of 0.43Å spanning 32 Cα atoms, superimposition of the active site of *Vd*PDA1 onto that of Pst_13661 indicates that both enzymes share a structurally similar substrate-binding pocket. Multiple sequence alignment of CE4 enzymes demonstrates a uniform distribution of conserved motifs and non-conserved insertions across the sequences of CE4 enzymes (Aragunde et al., 2018). The conserved motifs associated with enzyme activity are assigned numbers ranging from Motif 1 to Motif 5 and they are typically situated at the centre of the active site structure.

### 2.2 Active site conserved motifs

**Figure 2:**
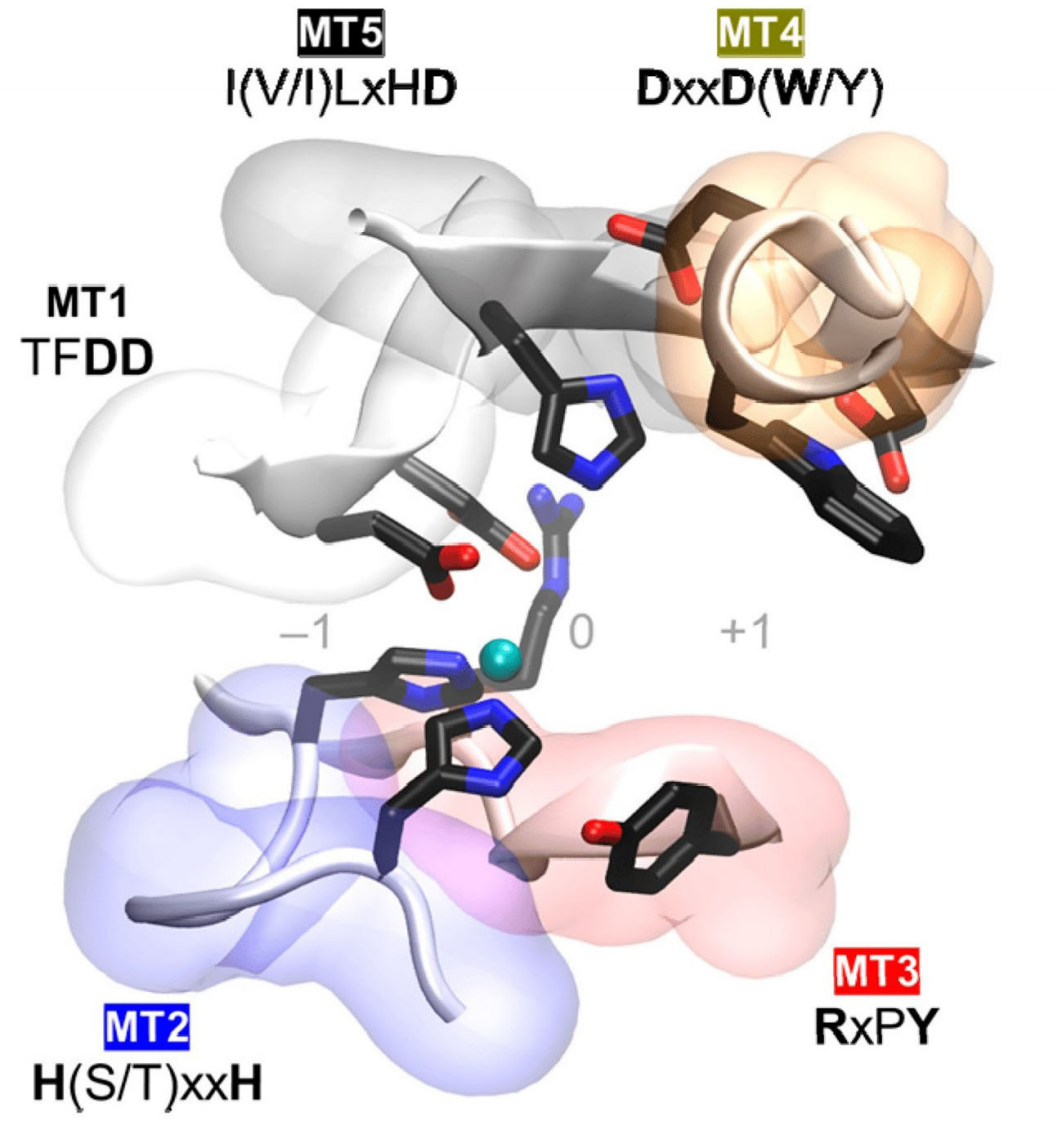
Conserved catalytic motifs, Motif 1 to Motif 5 (MT1–MT5) of plant pathogenic fungal CDAs and the spatial disposition in the 3D active site structure (Aragunde et al., 2018).

The NodB homology domain, spanning roughly 150 amino acids, is conserved among CE4 family members (Blair et al., 2005). The delineation of this particular region is commonly established based on the presence of five conserved motifs, which are denoted as Motif 1 to Motif 5 in accordance with their sequential arrangement within the sequence. These motifs constitute the active site and are necessary for the catalytic function of these enzymes. With the emergence of novel 3D structures, there is an opportunity to enhance the characterization of conserved motifs through the use of comprehensive sequence and structural alignments. Motif 1 (TFDD) has a significant degree of conservation. It has two aspartic acid (Asp) residues where one residue interacts with zinc or cobalt cation, and the other binds to the substrate to produce acetic acid. Motif 2 H(S/T)xxH is well recognized as a zinc-binding motif. In this motif, the two histidine (His) residues are responsible for binding the metal cation, while the serine (Ser) or threonine (Thre) residue forms a hydrogen bond with the second His, thereby stabilizing the loop-shaped motif’s conformation (Aragunde et al., 2018). The metal-binding Asp residue found in motif 1, in addition to the two His residues present in motif 2 are commonly referred to as the His-His-Asp metal-binding triad. Motif 3 (RxPY) is situated along one of the sides of the active site groove where it is an integral component, and it plays a crucial role in facilitating interactions with other residues inside the active site. It can bind acetic acid and zinc and coordinate the catalytic activity of Asp. Motif 4, characterized by the amino acid sequence DxxD(W/Y), constitutes the complementary region of the active site groove. The region encompasses a hydrophobic residue that is exposed to the solvent as well as a buried Asp residue, and tryptophan is the key amino acid of this motif (Aragunde et al., 2018; Huang et al., 2022). Motif 5 is characterized by the presence of the amino acid sequence I(V/I)LxHD), where a leucine residue is situated within the hydrophobic pocket responsible for accommodating the substrate’s acetate methyl group, with the general acid His residue that facilitates catalytic activity (Blair et al., 2005; Aragunde et al., 2018; Grifoll-Romero et al., 2018). The motif sequences of the characterized plant pathogenic fungal CDAs are shown in **Table 1** and the 3D structure of the spatial disposition of the active site is shown in **Figure 2**.

**Table 1:** Motif sequences for the characterized plant pathogenic fungal CDAs.

	Motif 1	Motif 2	Motif 3	Motif 4	Motif 5	Reference
***Cl*CDA**	TYDD	HTYAH	RAPY	DTKDY	IVLSHD	(Tokuyasu et al., 1996)
***Pes*CDA**	TFDD	HTWSH	RPPY	DTLDY	VSLMHD	(Cord-Landwehr et al., 2016)
***Pgt*CDA**	TYDD	HTWSH	RPPY	DSEDA	ITLNHE	(Figueroa et al., 2016)
**Cbp1**	TFDD	HTWSH	RPPY	DTEGY	LQIEHD	(Kuroki et al., 2017)
***Vd*PDA1**	TFDD	HTWDH	RPPY	DTNDW	IVLAHD	(Gao et al., 2019)
***Fov*CDA**	TFDD	HTYSH	RPPY	DSLDW	IALFHD	(Gao et al., 2019)
**Pst_13661**	TFDD	HTWSH	RPPY	DSGDT	ISLNHE	(Xu et al., 2020)
***Po*CDA7**	TFDD	HSWSH	RPPY	DSDDW	IVLAHD	(Dai et al., 2021)
***Ps*CDA2**	TFDD	HTWSH	RAPY	DSGDS	IALNHE	(Xiao et al., 2023)
***Fg*PDA5**	TFDD	HTYGH	RPPY	DTDDW	IVLHHD	(Hu et al., 2023)

## 3 Catalytic mechanism and substrate specificity of plant pathogenic fungal CDAs

### 3.1 Catalytic mechanism

The catalytic core of fungal CDAs and CE4 enzymes exhibits a remarkable degree of conservation among these enzymes despite their evolutionary divergence. The catalytic domains of the examined CDAs exhibit local variations in their physicochemical characteristics. Firstly, there are changes in the charge distribution inside the catalytic cavities and their surroundings. Secondly, the fungal CDAs have a greater level of hydrophobicity in the catalytic subsite +1. The unique characteristics of these enzymes, including their local distinct features and the length and flexibility of the loops that make up the catalytic cavities, may contribute to their specificity. This suggests that these enzymes can bind and process chitin oligomers that have been partly deacetylated, although with reduced efficiency, highlighting the significance of the acetyl group for the interaction with the subsite +1 throughout the chitin deacetylation process. The N-acetylglucosamine unit and some partly deacetylated oligomers have the ability to attach to the active sites of CDAs without causing a deacetylation process and this demonstrates their potential to block these enzymes (Roman et al., 2019). CDAs and their associated CE4 enzymes are typical metal dependent hydrolases. The catalytic mechanism of these enzymes resembles that of other hydrolases reliant on metal ions and is facilitated by the process of metal-assisted acid/base catalysis (Hernick and Fierke, 2005; Naqvi et al., 2016).

The presence of zinc cation inside the active site of *Cl*CDA suggests the significant involvement of metal cations in its function (Blair, 2006). The involvement of Zn^2+^, carbonyl group of Asp40, and the imidazole groups of His97 and His101 is essential for catalysis to occur in the catalytic step. Initially, the nucleophilic oxygen atom of the activated water molecule initiates an attack on the carbonyl carbon atom of the substrate. Simultaneously, D39, functioning as a general base undergoes proton absorption to generate a tetrahedral transition state intermediate with a negative charge. This intermediate is stabilized by the electrostatic contact between Zn^2+^ and the positively charged side chains of His97 and His101. Subsequently, the imidazole moiety of His295 functions as a general acid, donating a proton to the amino group (-NH) of the substrate while concurrently liberating acetate (Andrés et al., 2014). Additionally, Zn^2+^ is coordinated to the O7 atom of the N-acetyl group and the O3 hydroxyl of the GlcNAc ring. The water molecule achieves distorted octahedral coordination with the divalent metal cation. It was suggested that during activation, this particular water molecule acts as the nucleophile that is accountable for the elimination of the N-acetyl group (Andrés et al., 2014). *Vd*PDA1’s crystal structure revealed a metal ion coordinated to a signature Asp-His-His (formed by Asp56, His108, and His112) and a water molecule. The X-ray fluorescence spectra revealed the presence of two distinct peaks, corresponding to Ni^2+^ and Zn^2+^ ions, respectively, and it was also determined that, inside the active pocket, Zn^2+^ was coordinated by the His-His-Asp triad (Liu et al., 2023). The catalytic capacities of CDAs have been shown to be significantly influenced by divalent cations like Zn^2+^, Ca^2+^, Co^2+^, and Mg^2^, with the specific effect depending on the type of the substrate (Zhao et al., 2010). Moreover, a significant number of structures have acetate ions, which are formed as a result of the de-N-acetylation process (Roman et al., 2019). Previous studies have indicated that among the many divalent metal ions analyzed, Co^2+^ has the most advantageous effects on the enzymatic activity (Liu et al., 2017). Recently, kinetic analyses revealed that the increased activity of *Vd*PDA1 and Pst_13661 was due to their use of the Zn^2+^ ion rather than the Ni^2+^ ion. It was revealed that Asp55 and His201 of *Vd*PDA1 might serve as the catalytic base and catalytic acid, respectively, based on the catalytic processes of CE4 deacetylases (Andrés et al., 2014; Liu et al., 2023).

### 3.2 Substrate recognition and specificity

Fungal CDAs play a crucial role in various aspects of fungal biology, including nutrition, morphogenesis, and development (Ghormade et al., 2000; Zhao et al., 2010). These enzymes are involved in processes such as fungal autolysis, spore formation, cell wall formation and integrity, and germling adhesion (White et al., 2002; Matsuo et al., 2005; Baker et al., 2007; Geoghegan and Gurr, 2016). To effectively penetrate and colonize the host, the pathogen must be able to circumvent the host immune responses. Plants employ a defensive mechanism through the secretion of chitinases, enzymes that degrade the chitin present in the cell walls of fungi into smaller molecules known as chitooligosaccharides. These chitooligosaccharides are then detected by special receptors in plants that are designed to recognise chitin, thereby initiating a series of resistance responses (Sánchez-Vallet et al., 2015). However, plant fungal pathogens secrete CDAs throughout the process of infection and the initial stages of development inside the host (Ghormade et al., 2010). Accumulating evidence has reported successful pathogen evasion of host plant defense mechanisms through the partial deacetylation of the fungal cell wall chitin (El Gueddari et al., 2002; Liu et al., 2012; Sánchez-Vallet et al., 2015; Cord-Landwehr et al., 2016). Different fungal CDAs have distinct substrate modifications which depends on their specific roles within the fungal pathogen, presumably acting either in a processive or non-processive mode of action (Tsigos et al., 1999).

The fungal species *C. lindemuthianum*, belonging to the deuteromycete group, is responsible for the development of anthracnose, a plant disease that has significant economic implications for several crop species (Kauss and Bauch, 1988). *C. lindemuthianum s*ecretes a chitin deacetylase that exhibits activity towards both chitin oligomers and chitin polymers. The enzyme can completely remove acetyl groups from chitooligosaccharides that have a degree of polymerization (DP) of 3 or higher. However, it only removes acetyl groups from the non-reducing GlcNAc unit of N,N′-diacetylchitobiose (Tokuyasu et al., 1999; Tokuyasu et al., 2000a). In the case of substrates with a length exceeding DP3, a multiple-chain mechanism is observed. This mechanism follows a specific pathway in which the deacetylation process starts at the second residue from the reducing end (Tokuyasu et al., 2000a; Hekmat et al., 2003). The mono-deacetylation process in the beginning exhibits no discernible correlation between the catalytic rate constant (*k_cat_*) and the DP, but the Michaelis constant (*K_M_*) decreased as the DP increases (Hekmat et al., 2003; Blair, 2006). Nevertheless, the analysis of full deacetylation kinetics revealed a correlation between a rise in substrate DP and an increase in *k_cat_*, as well as the concurrent drop in *K_M_*, (Tokuyasu et al., 1996). The reversibility of this enzyme has been observed, since it demonstrates the ability to catalyze the acetylation of chitosan oligomers (Tokuyasu et al., 1999; Tokuyasu et al., 2000b; Kang et al., 2014). A chitin deacetylase *Pes*CDA from *Pestalotiopsis* sp. was found to effectively act on colloidal chitin and it was also established that it can act on chitosans with a higher degree of acetylation as well as on chitooligosaccharides (Cord-Landwehr et al., 2016). However, *Pes*CDA did not act on crystalline chitin. It was also established that this enzyme has a high activity on short oligomers (tetraacetylchitotetraose), but no activity was found on longer oligomers (Tsigos et al., 1999; Cord-Landwehr et al., 2016).

*Pst* is an obligate biotrophic plant fungal pathogen that causes stripe rust, which is one of the most detrimental wheat disease worldwide (Chen, 2005; Chen, 2017). *PstCDA* acts on several polymers including chitosans, colloidal chitin and glycol-chitin (Xu et al., 2020; Xiao et al., 2023). It was also found that Pst_13661 has a high chitin and cellulose affinity (Xu et al., 2020). *Ps*CDA2 was found to have a high affinity for chitin and A6 (K_d_ = 15.99 and 3.80 µM, respectively) and a minimal binding to chitosan and cellulose (Xiao et al., 2023). *Puccinia graminis* f. sp. *tritici* (*Pgt*) is also an obligate biotrophic plant fungal pathogen that causes wheat stem rust (Figueroa et al., 2016). *PgtCDA* has activity on several polymers, including colloidal chitin, glycol-chitin, and chitosans. Notably, its activity on chitosans is positively correlated with the degree of acetylation. Insoluble polymers such as α- or β-chitin do not exhibit reactivity in this context. It was revealed that tetraacetylchitotetraose serves as the nominal substrate for chitooligosaccharides, since the enzyme exhibits an inability to catalyze reactions on substrates of shorter length. The enzymatic deacetylation of tetramers to hexamers follows a sequential pattern, wherein the enzyme selectively removes acetyl groups from all GlcNAc units except for the final two on the non-reducing end [AA(D)n−2] a mechanism involving several chains (Naqvi et al., 2016). *Fusarium graminearum* is a hemibiotrophic plant fungal pathogen that causes fusarium head blight which is among the most devastating diseases affecting the global production of wheat and barley. It was recently found that *F. graminearum* secretes *Fg*PDA5 to enhance its pathogenicity and this polysaccharide deacetylase exhibited a notable binding affinity towards chitin and fungal hyphae. Additionally, *Fg*PDA5 demonstrated the ability to effectively remove acetyl groups from chitohexose molecules containing six GlcNAc moieties (A6) *in vitro* (Hu et al., 2023).

## 4 Biological roles of plant pathogenic fungal CDAs

The susceptibility of filamentous fungi to hydrolase attack is attributed to the compromised integrity of their cell walls, resulting in an expedited demise. In a more explicit manner, it can be said that the chitooligosaccharides that are liberated from the cell walls of fungal pathogens have a greater propensity to be recognized as elicitors, hence triggering the activation of basal immunity as a defense mechanism against the invasion of pathogens (Hamel and Beaudoin, 2010). Plant pathogenic fungal CDAs are involved in various biological process such as cell wall formation and integrity, germlings adhesion and appressorium differentiation as well as evasion of host defense mechanisms through suppression of chitin-triggered immunity.

### 4.1 Cell wall formation and integrity

The fungal cell wall maintains cellular integrity in the face of various environmental conditions to which phytopathogenic fungi are exposed (Latgé and Beauvais, 2014; Geoghegan and Gurr, 2016). Chitin is a polymer composed of long β-1,4-linked N-acetylglucosamine chains that functions by forming rigid microfibrils, which serve to preserve the hyphal structure and provide protection from potential host attacks. Chemical modification of the fungal cell wall chitin by deacetylation catalyzed by chitin deacetylases results in the formation of chitosan which accumulates in the cell wall of phytopathogenic fungal infection structures. Chitosan is polycationic, more hydrophilic, and amorphous compared to chitin, suggesting that deacetylation has a significant influence on the structure and physical properties of the fungal cell wall which in turn, affect fungal development and morphogenesis (Geoghegan and Gurr, 2016).

### 4.2 Germling adhesion and appressorium differentiation

The accumulation of chitosan in the germ tube and appressorium was confirmed using the crop pathogen and model organism *Magnaporthe oryzae* where it was found to be critical for appressorium differentiation and development (Fujikawa et al., 2009; Fujikawa et al., 2012). This indicates that chitosan mediates the adhesion of germlings to surfaces, which is fundamental for physical stimuli perception (Geoghegan and Gurr, 2016). The development of the appressorium necessitates high cell wall flexibility and such plasticity can be enabled by chitin deacetylation. *Magnaporthe oryzae* (*M. oryzae*) Chitin-Binding Protein 1 (*CBP1*) gene encodes a chitin-deacetylase enzyme which also plays a key role in the initiation stage of appressorium differentiation and development (Kuroki et al., 2017). *M. oryzae* has six CDA homologues, in addition to Cbp1; however, none of these homologues are deemed essential for the process of appressorium development. Chitosan localization at the fungal cell wall was detected by the use of OGA^488^, demonstrating that the conversion of chitin into chitosan, catalyzed by Cbp1, takes place at the cell wall of germ tubes throughout the process appressorium differentiation by *M. oryzae*. Collectively, these findings provide substantiation that the chitin deacetylase function of Cbp1 is important for the process of appressorium differentiation (Kuroki et al., 2017). It was also demonstrated that the cotton root pathogen *V. dahliae* secretes a chitin deacetylase (*Vd*PDA1) which alters chitin oligomers inside cotton plants (Gao et al., 2019). Furthermore, the study revealed that this enzyme is exclusively found at the interfaces between the fungus and its host. *Vd*PDA1 effectively inhibits the immune response triggered by chitooligosaccharides, hence preventing the reactive oxygen species (ROS) production, mitogen-activated protein kinases (MAPKs) phosphorylation, and expression of defense-related genes (Gao et al., 2019). It was also revealed that *Fusarium oxysporum* f. sp. *vasinfectum* augment its virulence, by secreting a CDA (*Fov*PDA1), resulting in cotton wilt disease (Gao et al., 2019).

The roles of PoCda7, a chitin deacetylase from *Pyricularia oryzae* were recently successfully elucidated (Dai et al., 2021). The results of the phenotype analysis demonstrated that the knockout mutant of ΔPocda7 did not exhibit any notable impact on fungal morphogenic development, specifically in relation to conidiation, germination, appressorial formation, and the cell wall composition of conidium and hyphae (Dai et al., 2021). However, it was observed that the mutant displayed sensitivity towards ROS. The presence of glycerols is essential for the development of enough turgor pressure in appressoria, which enables successful invasion of the host surface. The decreased appressorium turgor pressure and reduced appressorium-mediated invasion of ΔPocda7 resulted in a notable decline in the fungal pathogenicity inside the host plants (Dai et al., 2021). *Blumeria graminis* f. sp. *tritici* (*Bgt*) is a biotrophic fungal pathogen that causes wheat powdery mildew which is among the factors that continuously threaten global wheat production. *Bgt* secretes *Bgt*CDA1, a CDA which promotes *Bgt* invasion. Knockdown of *BgtCDA1* inhibited *Bgt* invasion. However, it was found that TiAP1 interacts with BgtCDA1 and inactivates the deacetylation role of BgtCDA1 leading to the induction of wheat defense responses to suppress *Bgt* invasive growth and penetration (Yang et al., 2022).

### 4.3 Suppression of chitin-triggered immunity

Phytopathogenic fungi evolved various defense mechanism to evade host defense responses. The recognition of chitin oligomers by plant receptors in the apoplast initiates the activation of the plant’s immune system. CDAs safeguard pathogenic fungal hyphae from lysis by secreted plant chitinases by converting the superficial chitin present in the cell wall of plant pathogenic fungi into chitosan. A chitin deacetylase *Pes*CDA from *Pestalotiopsis* sp. which is an endophytic fungus found in its host plant tissues was reported to be involved in the modification of chitin oligomers which helps the pathogen to evade recognition by host plant immunity (Cord-Landwehr et al., 2016). The pathogenic fungus *Ustilago maydis* is responsible for the development of smut disease in maize plants via the stimulation of extensive tumour growth, facilitating the proliferation of fungal hyphae and subsequent spore formation (Lanver et al., 2017). The interaction between *U. maydis* and maize at the interface is primarily regulated by a repertoire of fungal effector proteins. These proteins are triggered in a sequential manner after the first contact and colonization of maize by the fungus (Ökmen et al., 2018). *U. maydis* also has six functional CDAs and one pseudogene, which facilitate the conversion of chitin into chitosan to avoid detection by chitin receptors, hence enhancing its virulence and ensuring its survival (Rizzi et al., 2021). Out of the six genes that were shown to be active, five were glycosylphosphatidylinositol-anchored, whereas one was identified as encoding a secreted soluble enzyme. The secreted soluble Cda4 protein was suggested to be involved in the process of deacetylating the chitin surface layer and producing the chitosan layer that surrounds biotrophic hyphae. The simultaneous inactivation of all CDA genes was not effective, indicating that chitosan plays a crucial role in maintaining cell wall integrity (Rizzi et al., 2021). The regulation of chitin and chitosan biosynthesis is crucial due to their significant roles in fungal cell wall integrity, determination of fungal virulence, and serving as scaffolds for attachment to other wall components (Baker et al., 2007; Geoghegan and Gurr, 2016; Upadhya, 2018; Gao et al., 2019; Rizzi et al., 2021). Therefore, it is necessary to ensure that their production is appropriately controlled in response to environmental fluctuations, both in terms of spatial and temporal regulation.

It was found that the pathogen *Pgt* secretes *Pgt*CDA, an enzyme that facilitates the conversion of chitin to chitosan. This conversion is significant since it has been demonstrated that the invasive hyphae of *Pgt* are covered with chitosan rather than chitin (Naqvi et al., 2016). During infection, *Pst* secretes a chitin deacetylase Pst_13661 that enhances pathogen virulence by suppressing wheat chitin-triggered immunity. This implies that Pst_13661 has the potential to alter the composition of the fungal cell wall, hence impeding its detection by host plant apoplastic surveillance systems. Notably, enhanced resistance to three major *Pst* races CYR31, CYR32 and CYR33 was exhibited by transgenic wheat harboring the RNAi Pst_13661 construct. This resistance was achieved by impeding the growth and development of *Pst*, demonstrating that Pst_13661 is a key virulence factor and a promising target for the development of wheat breeding cultivars with broad-spectrum resistance. These findings unraveled novel strategies for the development of resilient germplasm that is resistant to diseases by disrupting fungal invasive growth and proliferation and this contributes to the development of innovative ways for managing plant fungal diseases (Xu et al., 2020).

Recently, another *Pst* CDA, *Ps*CDA2 was also found to enhance pathogen virulence by suppressing wheat chitin-triggered immunity (Xiao et al., 2023). *Ps*CDA2 expression was induced during the early stages of infection, and it also suppressed cell death in *N. benthamiana* leaves. *PsCDA2* silencing reduced pathogen virulence and enhanced wheat resistance against *Pst* infection, indicating the importance of *Ps*CDA2 during wheat-*Pst* interactions (Xiao et al., 2023). Furthermore, it was demonstrated that *Ps*CDA2 can safeguard *Pst* urediniospores against potential harm caused by the host chitinase *in vitro*. *Ps*CDA2 exhibited the ability to inhibit the host plant innate immune response induced by chitin, including reduction of callose deposition and expression of defense-related genes (Xiao et al., 2023). These findings indicate that *Pst* secretes *Ps*CDA2, a chitin deacetylase enzyme, which plays a crucial role in initiating infection and altering the acetyl group to hinder the degradation of chitin inside the cell wall by chitinases produced by the host plant. This elucidates a method by which the fungal pathogen inhibits the immune response of plants, therefore enhancing our comprehension of the management of wheat stripe rust. The ramifications of this knowledge are of considerable importance in the formulation of effective policies aimed at safeguarding crops against the detrimental impacts of this disease (Xiao et al., 2023).

In another recent study, seven polysaccharide deacetylases were identified from *F. graminearum* (Hu et al., 2023). It was found that, of the seven polysaccharide deacetylases, *FgPDA5* was a critical pathogenicity factor that was specifically expressed during pathogenesis. *FgPDA5* mutant reduced pathogen virulence by compromising fungal growth and the ability of the pathogen to infect wheat. The deacetylation of chitin oligomers by *Fg*PDA5 was shown to effectively inhibit the activation of plant defense mechanisms. *Fg*PDA5 showed the ability to effectively remove acetyl groups from chitohexose molecules containing six GlcNAc moieties (A6) *in vitro* but the activation of an autocatalytic defensive response was not observed when chitin oligomers were subjected to deacetylation by *Fg*PDA5. It was therefore speculated that *Fg*PDA5 potentially plays a positive role in the alteration of fungal chitin to evade detection by plant defense system. This discovery has the potential to provide a unique approach for disrupting hyphal remodeling, hence facilitating the development of robust breeding material with enhanced resistance to diseases (Hu et al., 2023). These findings demonstrated that *Fg*PDA5 is a polysaccharide deacetylase that enhances *F. graminearum* virulence by suppressing host plant chitin-triggered immunity in the apoplast via deacetylation of chitin oligomers (Hu et al., 2023).

## 5 Plant pathogenic fungal CDA inhibition

Significant progress has been achieved in the field of studying inhibitors that specifically target insect chitin-related enzymes, including chitinase (Chen and Yang, 2020). Nevertheless, the ubiquity of chitinases in microbes, plants, and people poses a challenge in the development of targeted pesticides. On the other hand, it is worth noting that CDAs are not present in humans or plants, therefore rendering inhibitors that specifically target CDAs as potentially effective environmentally friendly fungicides. The development of inhibitors targeting these crucial enzymes will contribute to a deeper understanding of their structural characteristics and underlying molecular mechanisms. There has been little progress in the development of inhibitors targeting CE4 enzymes, but with an optimistic outlook (Chen and Yang, 2020). Currently, there is a lack of available structural information about phytopathogenic fungal CDAs, which poses a challenge in developing strategies to effectively inhibit these enzymes (Liu et al., 2023).

Molecular topology (MT) integrated with quantitative structure-activity relationship (QSAR) is a very promising computer-aided drug design strategy for designing and discovering novel chemicals with targeted biological activity. The MT paradigm is based on chemo-mathematical descriptors which enable rapid and accurate prediction of several biological and physicochemical features (Zanni et al., 2015; Zanni et al., 2020; Zanni et al., 2022). MT is a branch of mathematical chemistry that focuses on the relationship between molecules and graphs, so it involves the use of graph theory indices to depict chemical structures (Galvez et al., 1994; Basak et al., 2000; García-Domenech et al., 2008; Carbó-Dorca, 2022). In addition, it focuses on the interconnection of atoms inside molecules rather than on geometric characteristics such as angles, distances, or three-dimensional structures, which are often addressed in traditional methodologies (Gálvez-Llompart et al., 2013; Zanni et al., 2015). MT has recently become the most common and effective plant protection approach used by researchers (Zanni et al., 2019; Galvez-Llompart et al., 2020). MT was also used to predict the fungicidal activity of various diphenylamine derivatives against three fungal species namely rice blast cucumber downy mildew, and cucumber grey mold (Zanni et al., 2019).

### 5.1 Categories of inhibitors

Fungicides are grouped based on their similarities in mode of action and chemical structure. Site-specific fungicides selectively interfere with certain metabolic processes or structural sites of the target fungus, such as cell division, sterol synthesis, or nucleic acid (DNA and/or RNA) production. Single or multiple-gene alterations may reduce the effectiveness of site-specific fungicides (Damicone, 2017). The site-specific methyl benzimidazole carbamate (MBC) fungicides interfere with cell division, demethylation inhibitor (DMI) fungicides disrupt synthesis of sterols, phenylamide (PA) fungicides inhibit fungal growth by disrupting RNA synthesis, quinone-outside inhibitor (QoI) fungicides target the mitochondrial protein cytochrome bc-1 to inhibit respiration in fungal cells, and succinate dehydrogenase inhibitor (SDHI) fungicides inhibit respiration in fungal pathogens by blocking a fundamental mitochondrial enzyme (Ghannoum and Rice, 1999; Damicone, 2017). There is slower shift of resistance to these fungicides towards insensitivity due to multiple-gene involvement. The fungicides that target many sites interfere with multiple fungal metabolic processes and are mostly protectant fungicides, for example, dicarboximides that inhibit both spore germination and fungal growth. They have low risk of resistance (Damicone, 2017). The fungal chitin deacetylase inhibitors are site specific fungicides and so far, the only available inhibitors of these fungal CDAs were developed from specific carboxylic acids, particularly ethylenediamine tetraacetic acid (EDTA) (Zanni et al., 2019; Martínez Cruz et al., 2021), benzohydroxamic acid (BHA) and its derivatives (Liu et al., 2023), as well as various diphenylamine derivatives against three fungal species namely rice blast cucumber downy mildew, and cucumber grey mold (Zanni et al., 2019).

### 5.2 Significance of fungal CDA inhibitors

Previous studies on CDA in *Colletotrichum* sp. and other fungi have shown that the presence of specific carboxylic acids, specifically EDTA, leads to a reduction in enzyme activity *in vitro* (Araki and Ito, 1975; Kafetzopoulos et al., 1993; Christodoulidou et al., 1999; Yamada et al., 2008; Pacheco et al., 2013; Zanni et al., 2019; Martínez Cruz et al., 2021). It was demonstrated that CDA inhibitors such as carboxylic acids reduced the severity of powdery mildew and induce rapid activation of chitin-triggered immunity, indicating that CDA could be an interesting target for the development of fungicides (Zanni et al., 2022). Furthermore, as proposed by previous researchers, the negative effect of carboxylic acids may be attributed to a negative feedback mechanism induced by acetate, the precursor of carboxylic acid (Pacheco et al., 2013). Intriguingly, the application of EDTA triggered a plant defense mechanism that resembled the reaction achieved by the suppression of *Px*CDA, a *Podosphaera xanthii* CDA (Martínez Cruz et al., 2021). Experiments were performed to examine the potential association between this host response and chitin signaling by using plants with silenced CERK1 and treating them with EDTA. The study found that when the fungal CDA and plant chitin receptors were co-silenced in plants, there was a partial but significant restoration of fungal growth (Martínez Cruz et al., 2021). This supports the hypothesis that the inhibition of CDA activity by EDTA can activate chitin signaling by interfering with the chitin deacetylation process (Gao et al., 2019).

It was also found that EDTA can effectively stop the progression of diseases caused by necrotrophic fungi *Botrytis cinerea* and *Penicillium digitatum*. The disease-suppression action of EDTA was independent of its chelating activity since other chelating agents did not exhibit disease suppression. In addition, EDTA effectively inhibited the growth of green and grey mold-causing pathogens when applied to oranges and strawberries, respectively. These findings provide strong evidence that CDA is a very viable target for managing phytopathogenic fungi, suggesting that EDTA might serve as a scaffold for the design of novel fungicides (Martínez Cruz et al., 2021). Recently Zanni and colleagues discovered novel, specific and potent CDA inhibitor using an *in silico* strategy based on QSAR and MT (Zanni et al., 2022). Three predictive equations based on the MT paradigm were developed to identify a set of candidate molecules, starting with the chemical structures of a few carboxylic acids with and without disease control activities. Their fungicidal efficacy was examined experimentally, and their specificity as CDA inhibitors was investigated using molecular docking simulations for the three best candidates. This is the first time MT has been utilized to identify prospective CDA inhibitors for use against resistant powdery mildew strains. In this regard, it is intriguing to discover molecules capable of stimulating plant immune systems by inducing a defensive response against phytopathogenic fungi that are extremely resistant to fungicides, such as powdery mildew (Zanni et al., 2022).

**Figure 3:**
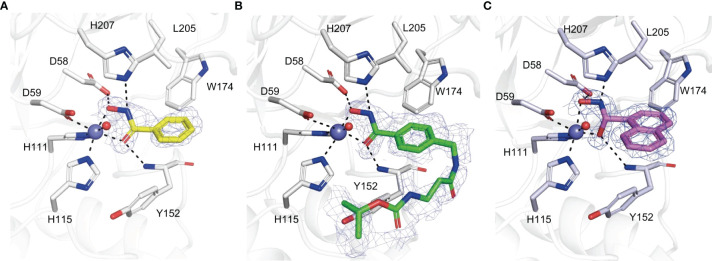
**(A–C)** Amino acid residues involved in the binding of BHA **(A)**, BHA derivative 1 **(B)**, BHA derivative 2 **(C)** within the active site of Pst_13661. BHA, BHA derivative 1, and 2 are shown as sticks with yellow, green, and light pink carbon atoms, respectively. The Pst_13661 residues that interact with these compounds are shown as sticks with blue carbon atoms. Dashed lines indicate hydrogen bonds. Slate and red spheres indicate the zinc and water molecules, respectively. The amino acids are labeled by their one-letter abbreviations (Liu et al., 2023).

BHA is a typical chelator of metal ions like zinc and nickel and is also an inhibitor of many metalloprotein enzymes such as histone deacetylases, ureases, carbonic anhydrases and matrix metalloproteases (Vreese and D’hooghe, 2017). Four compounds with a BHA moiety were revealed to be phytopathogenic fungal chitin deacetylase inhibitors, with BHA being the most effective one (Liu et al., 2023). To demonstrate the metal ion chelation properties of BHA in relation to CDAs, Pst_13661 crystal structure in complex with BHA, and other two BHA derivatives were successfully determined. BHA exhibits bidentate coordination by chelating a zinc ion (**Figure 3A**). The benzene ring moiety of BHA is accommodated inside a cavity that is constituted by the nonpolar residue Leu205, as well as the aromatic residues Tyr152 and Trp174. The inhibitory mechanism of BHA has resemblance to the inhibitory mechanism observed in the structures of Pst_13661 when bound to other two BHA derivatives. These structures indicate that the hydroxamic acid moiety plays a crucial role in inhibition by forming a chelation complex with the zinc ion, which is catalytically significant, and interacting with the catalytic residues (**Figures 3B, C**) (Liu et al., 2023).

BHA showed considerable efficacy in managing the severity of plant diseases caused by plant pathogenic fungi. Although the application of BHA doesn’t affect the growth and proliferation of these fungal pathogens, it caused a considerable reduction in the number of lesions and fungal biomass in soybean hypocotyls infected with *F. oxysporum, F. graminearum* or *Rhizoctonia. solani*. Furthermore, the disease indices exhibited a significant decrease after the infection of wheat plants with *Pst*, when treated with different doses of BHA. Therefore, it is plausible that BHA functions as an activator of plant immunity and may not specifically target the pathogenic fungi. Nevertheless, it was proposed that BHA might serve as an inhibitor of some metalloproteins that enhance the evasion of the host’s defense responses and there is still need for further research to explore other viable mechanisms involved (Liu et al., 2023). The primary objective of fundamental research on pathogenesis is to mitigate the sternness incidence of plant fungal diseases or ameliorate their austerity while enhancing production practices. The application of BHA might, however, have some side effects on crop protection, which still needs further exploration of its limitations. The discovery of BHA as an inhibitor of phytopathogenic CDAs will undoubtedly lead to the rational design of inhibitors that target pathogenic fungal CDAs, based on the BHA scaffold. This notion was previously unknown to both plant protection researchers and agrochemical designers. Therefore, it is possible that the management of plant fungal diseases might be achieved by the identification of novel antifungal targets of chitin deacetylases, chitosan, or other significant virulence factors.

### 5.3 Risk assessment

Assessment guides the formulation of effective resistance management techniques and determines the level of monitoring required. Understanding the method by which a fungicide works may provide valuable information. To accurately evaluate the likelihood of resistance development in a specific area, it is crucial to consider and incorporate all significant factors such as the inherent risk associated with each combination of fungicide and pathogen, the impact of environmental conditions on disease occurrence, and the relevant agricultural practices (Brent and Hollomon, 2007). It is important to incorporate any specific fungicide use strategies recommended by the manufacturer. Undoubtedly, such risk assessment can only provide a rough estimation, at most suggesting a low, medium, or high level, due to the involvement of several factors. The development of resistance and its accumulation in the field is influenced by several variables, making the prediction of resistance risk for novel fungicides challenging. Although resistance issues have been observed after the introduction of several new fungicides, there are still many instances when their efficacy remains intact. When evaluating resistance risk, it is important to consider many factors such as the characteristics of the fungicide, the biology of the pathogen, and the specific crop production system in which the fungicide is applied. The registration labels of most site-specific fungicides now prominently include information on the mode of action group and resistance management techniques. Nevertheless, accurately predicting the precise likelihood of resistance is challenging due to the complex interplay of several elements. It is crucial to monitor the levels of resistance in pathogen populations for risk assessment and evaluation of the effectiveness of management strategies. Unfortunately, there is currently no coordinated system for monitoring in place, and producers will often need to depend on established techniques for managing resistance.

### 5.4 Limitations and future prospects

The invention of CDA inhibitors is critical for the agricultural sectors and more specifically to the agrochemical industries because it avails probable plant protection tools against phytopathogenic fungi that continuously threaten global food security. Furthermore, the discovery of novel fungicide targets is of great significance due to the increasing prohibition of various active ingredients, leading to a decline in the availability of fungicides with unique action mechanisms. Hence, it is essential to expedite the development of novel agrochemicals. The indiscriminate use of fungicides has resulted in health and environmental issues connected with chemical residues and the selection of resistant diseases, leading to the implementation of increasingly stringent fungicide laws. Furthermore, the costs and timeframe for developing and commercializing novel fungicides are so high that only large crops often attract the requisite agrochemical-industry investment. The consensus is that limiting the variety of approved fungicides may result in the fast emergence of resistance to the remaining fungicides. It is well acknowledged that the prevention of fungicide resistance requires the use of chemistries that have three distinct mechanisms of action (Oliveira Garcia et al., 2021).

The prohibition of plant-protecting chemicals in agriculture provides a compelling case for the urgent need of developing novel fungicides with unique mechanisms of action to preserve a potent range of chemical options for effectively managing destructive diseases (Aliyeva-Schnorr et al., 2023). It is crucial to carefully evaluate their selectivity in order to minimize potential harm to non-target species, such as humans. Therefore, there is an urgent need for crystal structure information on CDAs from different phytopathogenic fungi. Another concern that requires more attention is the druggability and off-target impact, necessitating further efforts for improvement. In the future, it is anticipated that the integration of advanced computational approaches, with emerging artificial intelligence (AI) technologies, will play a significant role in enhancing the druggability and create novel scaffolds of compounds for use as novel fungicides (Chen and Yang, 2020). Furthermore, there is a great potential for the integration of molecular biology and MT for the rational discovery of new agrochemicals (Zanni et al., 2022). Although the EDTA molecule has the potential to be a promising candidate for the development of fungicides, the amounts that were deemed fungicidal were rather too high for practical use (Martínez Cruz et al., 2021). Therefore, there is need to carefully evaluate the selectivity of the appropriate concentrations to minimize potential harm to non-target species. Fungicides often target the fungal cell wall. Chitin production is seen as a secure target for fungicides since chitin is found in fungi and arthropod exoskeletons, but not in plants and mammals (Gow et al., 2017). There is currently just a single commercially available fungicide known as polyoxin D, which is a peptidyl pyrimidine nucleoside, that specifically targets the process of chitin synthesis by inhibiting the activity of chitin synthase (Endo et al., 1970). Hence, the suppression of chitin deacetylase would serve as a novel mechanism and an additional means to manage powdery mildews and other phytopathogenic fungi.

## 6 Conclusion

Chitin/polysaccharide deacetylases belong to the CE4 enzyme family, and they have a 3D fold characterized by a (β/α)_8_ structure. These enzymes are classified as metal-dependent hydrolases, with Zn^2+^ or Co^2+^ being the predominant metal cations associated with their catalytic activity. They are also characterized by the conservation of five active site motifs, which encompass the His-His-Asp metal binding triad, as well as the catalytic Asp and His residues that serve as general base and general acid, respectively. Phytopathogenic fungal CDAs are key virulence factors that enhance pathogenicity through the deacetylation of chitin into chitosan which is a poor substrate for host plant chitinases. Therefore, CDAs enhances pathogen virulence by suppressing chitin-triggered immunity. Considering the significant functions of CDAs in biological attack and defense systems, they have potential uses in agriculture for controlling fungal plant pathogens. The discovery of new CDAs with distinct specificities and the resolution of the structures of new enzyme-substrate complexes will provide more understanding of the structure-specificity relationships.

## Author contributions

JM: Conceptualization, Writing – original draft, Writing – review & editing. JC: Writing – review & editing. HL: Writing – review & editing. YZ: Writing – review & editing. RL: Writing – review & editing. LS: Writing – review & editing. NZ: Writing – review & editing. WY: Funding acquisition, Writing – review & editing.
